# Decoding and geometry of ten finger movements in human posterior parietal cortex and motor cortex

**DOI:** 10.1088/1741-2552/acd3b1

**Published:** 2023-05-25

**Authors:** Charles Guan, Tyson Aflalo, Kelly Kadlec, Jorge Gámez de Leon, Emily R Rosario, Ausaf Bari, Nader Pouratian, Richard A Andersen

**Affiliations:** 1 California Institute of Technology, Pasadena, CA, United States of America; 2 T&C Chen Brain-Machine Interface Center at Caltech, Pasadena, CA, United States of America; 3 Casa Colina Hospital and Centers for Healthcare, Pomona, CA, United States of America; 4 David Geffen School of Medicine at UCLA, Los Angeles, CA, United States of America; 5 University of Texas Southwestern Medical Center, Dallas, TX, United States of America

**Keywords:** finger decoding, hand movement, brain-computer interface (BCI), posterior parietal cortex (PPC), motor cortex (MC), representational geometry, factorized representations

## Abstract

*Objective*. Enable neural control of individual prosthetic fingers for participants with upper-limb paralysis. *Approach*. Two tetraplegic participants were each implanted with a 96-channel array in the left posterior parietal cortex (PPC). One of the participants was additionally implanted with a 96-channel array near the hand knob of the left motor cortex (MC). Across tens of sessions, we recorded neural activity while the participants attempted to move individual fingers of the right hand. Offline, we classified attempted finger movements from neural firing rates using linear discriminant analysis with cross-validation. The participants then used the neural classifier online to control individual fingers of a brain–machine interface (BMI). Finally, we characterized the neural representational geometry during individual finger movements of both hands. *Main Results*. The two participants achieved 86% and 92% online accuracy during BMI control of the contralateral fingers (chance = 17%). Offline, a linear decoder achieved ten-finger decoding accuracies of 70% and 66% using respective PPC recordings and 75% using MC recordings (chance = 10%). In MC and in one PPC array, a factorized code linked corresponding finger movements of the contralateral and ipsilateral hands. *Significance*. This is the first study to decode both contralateral and ipsilateral finger movements from PPC. Online BMI control of contralateral fingers exceeded that of previous finger BMIs. PPC and MC signals can be used to control individual prosthetic fingers, which may contribute to a hand restoration strategy for people with tetraplegia.

## 1. Introduction

Tetraplegic individuals identify hand function as a high-impact priority for improving their quality of life [1–3]. Neuroprosthetics research has enabled control of basic grasp shapes [4–7], an important step towards empowering paralyzed individuals to perform daily activities. However, these predefined grasp templates constrain the range of motion and thus limit the usefulness of existing neural prosthetics.

The complexity of human motor behavior is largely enabled by our versatile, dexterous hands [8]. The human hand can weave intricate crafts, sign expressive languages, and fingerpick guitar solos. Even everyday manual behaviors, like turning a door handle, require volitional control over many degrees of freedom [9]. Indeed, humans can move individual fingers much more independently than other animals, including monkeys [10, 11]. To better restore autonomy to people with tetraplegia, neural prosthetics would benefit from enabling dexterous finger control.

Intracortical brain–machine interface (BMI) research has largely focused on control of computer cursors and robotic arms, rather than dexterous hand control. Building off foundational studies of non-human primates [12–18], several clinical studies have implemented continuous decoders for cursor control [19–23]. Leveraging this cursor control, subsequent studies [24–26] developed on-screen keyboard typing interfaces for tetraplegic participants. [5–7, 27] extended continuous decoding to arm control, with [27] controlling the user’s own muscles. Recent work has also decoded speech from sensorimotor cortex [28–31]. However, relatively few BMI studies have focused on hand control [32–37], and previous studies frequently combine the ring and little fingers or leave them out altogether. Individuated finger control would be useful for applications like keyboard typing or object manipulation.

Most motor BMIs record neural activity from the MC, although areas of the posterior parietal cortex (PPC) have also been used successfully for BMI control of reaching [15, 22] and grasping [4]. The PPC plays a central role in sensorimotor integration, with regions of PPC representing visual stimulus locations and eye movements [38], task context [39], planned reaches [40], and object grasping [41, 42]. PPC uses partially mixed selectivity to simultaneously encode many motor variables [43], which can be useful for versatile neural decoding.

Despite PPC’s clearly demonstrated role in grasping [8, 42, 44], less is known about PPC responses during individual finger movements. With functional magnetic resonance imaging (fMRI), lesion, and anatomical evidence situating primary MC as core to fine finger movements (for review, see [8]), most electrophysiological studies of finger movements have focused on the primary motor and primary somatosensory cortex [33, 34, 45–50]. Nevertheless, non-human primate mapping studies [51] and stimulation studies [52, 53] have identified PPC sub-regions that are likely involved in fine finger movements. These results imply that fine finger movements are supported by a broad neuronal network, which should be investigated to improve dexterous BMI control.

Here, we recorded intracortical activity from the PPC of two tetraplegic participants while they attempted to press individual fingers. Across task contexts, we could classify individual finger movements during planning and attempted-execution periods. We connected this neural decoder to drive a neural prosthetic hand, with accuracies exceeding recent intracortical BMI studies [36, 54]. Furthermore, we characterize both the neural tuning and representational geometry [55] during attempted finger movements of either hand. The neural code factorized into finger type and laterality components, leading to finger representations that were simultaneously discriminable and similar across contralateral/ipsilateral pairs of fingers. These findings contribute to the understanding of human hand movements and advance the development of hand neuroprosthetics for people with paralysis.

## 2. Methods

### 2.1. Study participants

Experiments were conducted with two volunteer participants enrolled in a BMI clinical study (ClinicalTrials.gov Identifier: NCT01958086). All procedures were approved by the respective institutional review boards of California Institute of Technology, Casa Colina Hospital and Centers for Healthcare, and University of California, Los Angeles. Each participant consented to this study after understanding the nature, objectives, and potential risks.

Participant NS is a right-handed, tetraplegic woman. Approximately ten years before this study, she sustained an AIS-A spinal cord injury at cervical level C3-C4. NS can move her deltoids and above, but she cannot move or feel her hands.

Participant JJ is a right-handed, tetraplegic man. Approximately three years before this study, he sustained a spinal cord injury at cervical level C4-C5. He has residual movement in his upper arms, but he cannot move or feel his hands.

Because both participants could not move or feel their hands, we instructed them, during the behavioral tasks, to attempt finger movements as if their fingers were not paralyzed. We often abbreviate these finger movement attempts as ‘finger movements.’

### 2.2. Tasks

#### 2.2.1. Alternating-cues finger press task with delay

**Figure 1.**
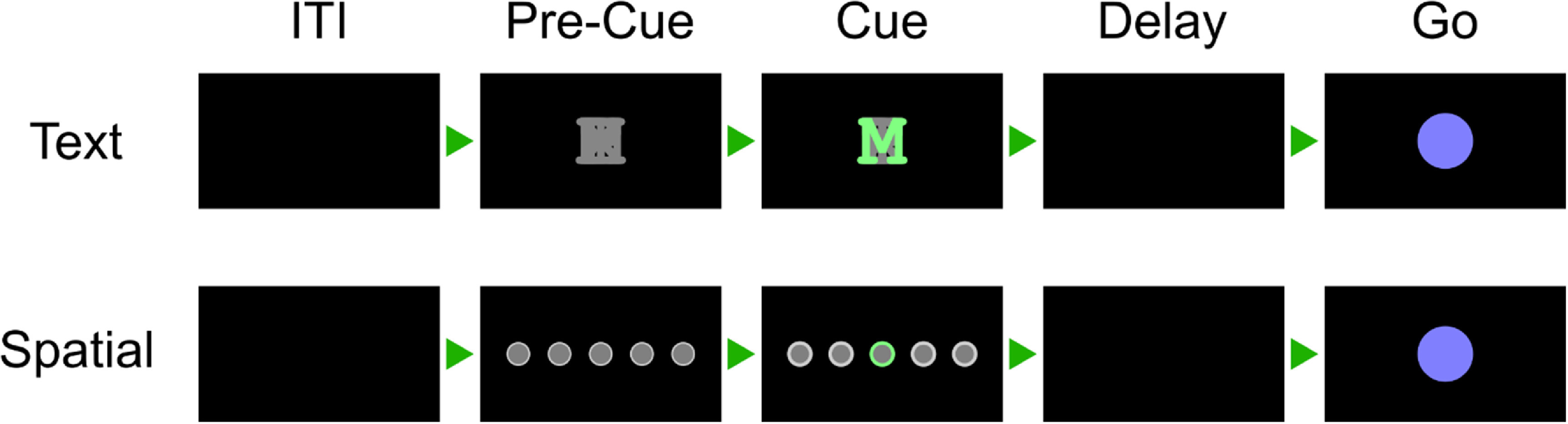
Alternating-cues, instructed-delay finger press task. Trial structure. Each rectangle represents the computer monitor display at each phase. Two cue variants, text and spatial, were trial-interleaved. In the spatial variant, the location of the highlighted circle corresponded to the cued finger. Trials without a highlighted circle indicated a No-Go cue. In the text variant, a highlighted letter (for example, ‘M’ for the middle finger) cued each finger. In both variants, the finger cue disappeared before the movement phase (Go) to separate planning and execution periods. Phase durations are listed in supplementary table 1.

Each participant performed an instructed-delay finger movement task (figure 1). They were seated in front of a computer monitor display, with their hands prone on a flat surface. Each trial began with a cue specifying a finger of the right hand. The finger cue then disappeared during a delay period. A cue-invariant go-icon appeared, instructing the participant to attempt to press the cued finger as though pressing a key on a keyboard. This instructed-delay task format temporally separates the visual stimulus from the planning and execution epochs.

Supplementary table 1 documents the phase durations for each task, and supplementary table 2 lists the date ranges for each task.

Some regions of the PPC are modulated by non-motor variables like visual stimulus location [38] and task context [39]. To ensure that the recorded neural signals reflected movement type (rather than, e.g. visual memory), we varied the cueing method between runs (figure 1). In the Spatial-Cue variant, five circles corresponded to the five fingers. In the Text-Cue variant, the finger cue was a letter abbreviation. A brief Pre-Cue phase in each trial indicated what cue-variant the trial would be.

#### 2.2.2. Finger press task with randomized cue location (reaction-time)

**Figure 2.**
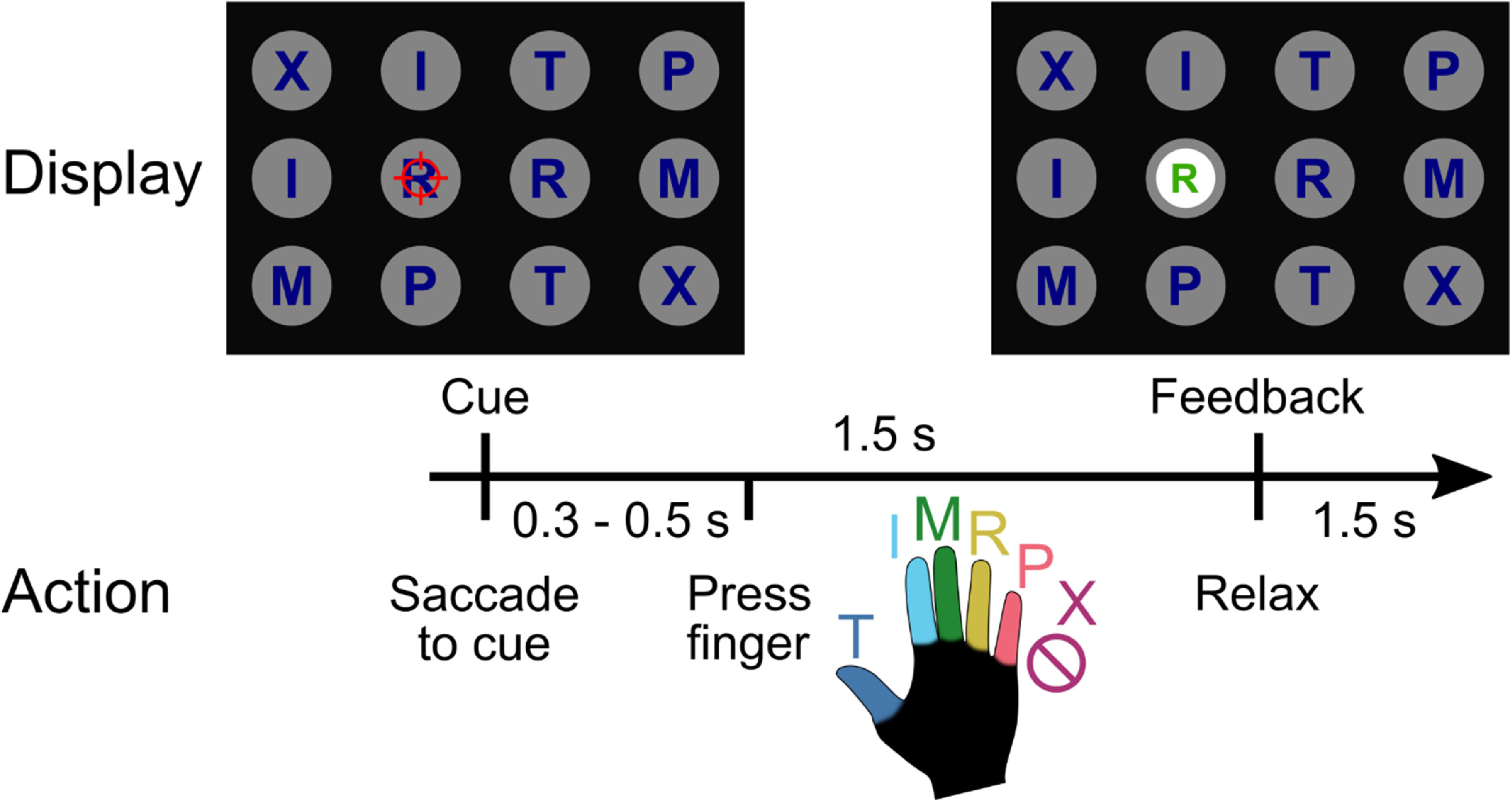
Reaction-time finger-press task with randomized cue location. When a letter was cued by the red crosshair, the participant looked at the cue and immediately attempted to flex the corresponding digit of the right (contralateral) hand. We included a No-Go condition ‘X’, during which the participant looked at the target but did not move their fingers. Visual feedback indicated the decoded finger 1.5 s after cue presentation. To randomize the saccade location, cues were located on a grid (three rows, four columns) in a pseudorandom order. The red crosshair was jittered to minimize visual occlusion. Reproduced from [54]. CC BY 4.0.

Letters, corresponding to each movement type, were arranged in a 3 × 4 grid across the screen (figure 2). Each grid consisted of two repetitions each of T (thumb), I (index), M (middle), R (ring), P (pinky), and X (No-Go). Letters were arranged in a random order to dissociate eye gaze signals from movement representations. On each trial, a single letter cue was indicated with a crosshairs symbol, which was jittered to minimize systematic effects of letter occlusion. Each cue was selected once (for a total of 12 trials) before the screen was updated to a new arrangement. Each run-block consisted of four screens for a total of 48 trials.

On each trial, the participant was instructed to immediately saccade to the cued target and fixate, then attempt to press the corresponding finger of the right hand. A trained classifier decoded the finger movement from neural signals and displayed the classified finger movement 1.5 s after the start of the trial. The participant pressed the instructed finger and fixated on the cue until the visual classification feedback was shown.

Data from participant NS performing this task was previously analyzed in [54]. Data from participant JJ have not been reported previously. During three sessions, participant JJ also performed this task using his left hand.

#### 2.2.3. Ten-finger press task

**Figure 3.**
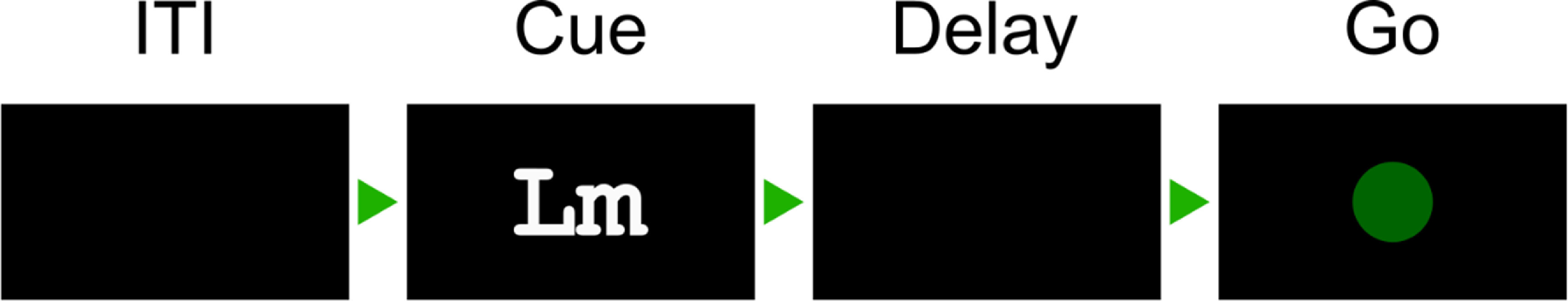
Text-cued finger movement task with instructed-delay. Trial structure. Text cues indicate the hand (‘R’ or ‘L’) and the finger (e.g. ‘m’ for middle finger). After a delay period, a cue-invariant Go-icon instructs movement execution.

Each participant also performed an instructed-delay finger press task with fingers from both hands. The task was like the Text-Cue variant of the Alternating-cues finger press task with delay, except without a Pre-Cue phase. All ten fingers were interleaved in trials within the same run-block (figure 3). Phase durations are documented in supplementary table 1.

### 2.3. Implant location

Participant NS was implanted with two 96-channel NeuroPort Utah electrode arrays six years after injury (about four years before this study). The implant locations were determined using anatomical priors and preoperative fMRI [54]. One array (denoted NS-PPC) was implanted over the hand/limb region of PPC at the junction of the intraparietal sulcus (IPS) with the postcentral sulcus. This region is thought to be involved in the planning of grasp movements [4, 42, 56]. In this report, we refer to this brain area as PC-IP (postcentral-intraparietal), although it is sometimes also referred to as the anterior IPS region [57]. A second array was in Brodmann’s area (BA) 5d. In the weeks following implantation, it was found that the BA 5d array did not function, so only the PC-IP array was used in this study.

Participant JJ was implanted with two 96-channel NeuroPort Utah electrode arrays about 20 months after injury (about 35 months before this study). The first array (denoted JJ-PPC) was implanted in the superior parietal lobule (SPL) of the left PPC. The second array (denoted JJ-MC) was implanted near the hand knob of the left MC (supplementary figure 1). PPC and MC activity were recorded simultaneously.

### 2.4. Neural signal recording and preprocessing

Neural signals were acquired, amplified, bandpass-filtered (0.3 Hz–7.5 kHz) and digitized (30 kHz, 16 bits/sample) from the electrodes using NeuroPort Neural Signal Processors (Blackrock Microsystems Inc.).

Action potentials (spikes) were detected by high-pass filtering (250 Hz cut-off) the full-bandwidth signal, then thresholding at −3.5 times the root-mean-square voltage of the respective electrode. Although one or more source neurons may generate threshold crossings, we used raw threshold crossings for online control and only sorted spikes for offline analyses. Single neurons were identified using the k-medoids clustering method. We used the gap criteria [58] to determine the total number of waveform clusters. Clustering was performed on the first *n* ∈ {2, 3, 4} principal components, where *n* was selected to account for 95% of waveform variance.

### 2.5. Feature extraction

Except when otherwise specified, we used a 500 millisecond (ms) window of neural activity to calculate firing rates (counted spikes divided by the window duration). The firing rate was then used as the input features to each analysis or classification model.

For cross-validation classification analyses, neurons with an average firing rate on the training fold <1 Hz were discarded as noisy features. For single-neuron analyses, a looser threshold of <0.5 Hz, averaged over the entire recording, was used to exclude neurons from significance and effect size tests.

Behavioral epochs: the movement execution (‘Go’ or ‘move’) analysis window was defined as the 500 ms window starting 200 ms after the Go cue. For applicable tasks, the movement planning (‘Delay’ or ‘plan’) analysis window was defined as the 500 ms window starting 200 ms after the Delay screen. The cue analysis window was defined as the 500 ms window starting 200 ms after the cue screen. The intertrial interval (ITI) analysis window was defined as the last 500 ms of the ITI phase.

### 2.6. Single-neuron selectivity for finger movements

In the section ‘Single-neuron modulation to individual finger presses’, we used a one-way ANOVA to determine whether neurons distinguished firing rates between different attempted finger movements. A neuron was considered discriminative if *p* < 0.05 after false discovery rate (FDR) correction for multiple comparisons using the Benjamini–Hochberg procedure; we also denoted this FDR-adjusted *p*-value as *q*. We corrected for *m*= *N* comparisons, where *N* is the number of neurons for each participant. Following Cohen’s rules of thumb [59], we denoted the ANOVA effect size as ‘large’ if *η* ^2^ > 0.14. As the ANOVA post hoc test, we used Dunnett’s multiple comparison test [60] to determine which fingers had significantly different firing rates than the No-Go baseline.

**Figure 4.**
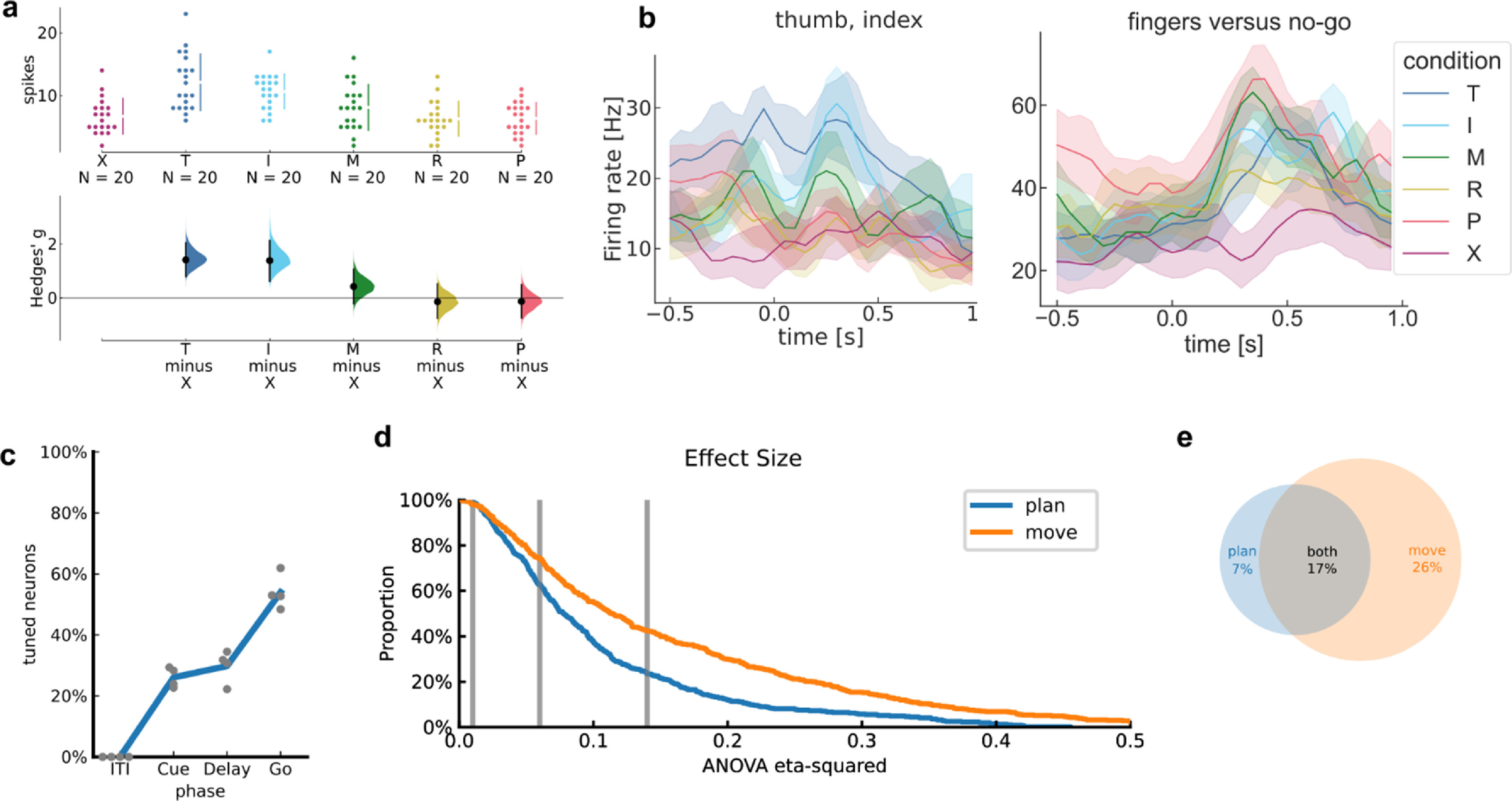
PPC single neurons discriminate between attempted finger movements. (a) Single-trial firing rates for an example NS-PPC neuron during attempted movements of different fingers. (top) Markers correspond to the firing rate during each trial. Gapped vertical lines to the right of markers indicate ± S.D., and each gap indicates the mean firing rate. (bottom) Firing rates during thumb (T) and index (I) presses were higher than the No-go (X) baseline. Vertical bars indicate bootstrap 95% confidence intervals (CI) of the effect size versus No-go baseline. Half-violin plots indicate bootstrap distributions. (b) Mean smoothed firing rates for each finger movement for two example NS-PPC neurons, which respectively modulated for thumb/index movements (left) and fingers versus No-Go (right). Shaded areas indicate 95% CI. (c) Percentage of NS-PPC neurons that discriminated between finger movements in each analysis window (*q* < 0.05, FDR-corrected for 466 neurons). Line (blue) indicates mean across sessions. Markers (gray) indicate individual sessions. (d) Complementary empirical cumulative distribution function visualizing the proportion of NS-PPC neurons with ANOVA effect sizes (*η* ^2^) above the corresponding *x*-axis value. Line colors indicate analysis epoch. Vertical lines (gray) indicate Cohen’s thresholds [59] for small (*η* ^2^ = 0.01), medium (*η* ^2^ = 0.06), and large (*η* ^2^ = 0.14) effect sizes. (e) Overlap of NS-PPC neurons that modulated significantly (*q* < 0.05) with large effect sizes (*η* ^2^ > 0.14) during movement preparation (plan) and movement execution (move).

To quantify the effect size of firing-rate changes against the No-Go baseline (figure 4(a)), we used Hedges’ g, which is similar to Cohen’s d but bias-corrected for small sample sizes. We calculated and visualized Hedges’ g values using the data analysis using Bootstrap-Coupled Estimation Python library [61].

For visual simplicity, we pooled neurons across sessions when calculating and visualizing single-neuron metrics (percentage selective, number of fingers discriminable from No-Go, empirical cumulative distribution functions).

To visualize firing rates, spike rasters were smoothed with a Gaussian kernel (50 ms standard-deviation [S.D.]), then averaged across trials to create a peristimulus time histogram.

### 2.7. Offline classification with cross-validation

We trained a separate linear classifier for each session to predict attempted finger movements from the neural features. We used diagonal-covariance linear discriminant analysis (diagonal LDA) [62]; diagonal LDA is equivalent to Gaussian Naive Bayes (GNB) when GNB shares a single covariance matrix across classes.

To calculate aggregate classification accuracies, confusion matrices, and parameter sweeps, we first calculated the respective metrics for each session separately, using stratified K-folds cross-validation (*K* = 8, no shuffling) within each session. We then aggregated results across sessions by dividing the number of correct trials (summed across sessions) by the number of total trials (summed across sessions). Across-session standard deviations of classification accuracy are weighted by the number of trials in each session.

**Figure 5.**
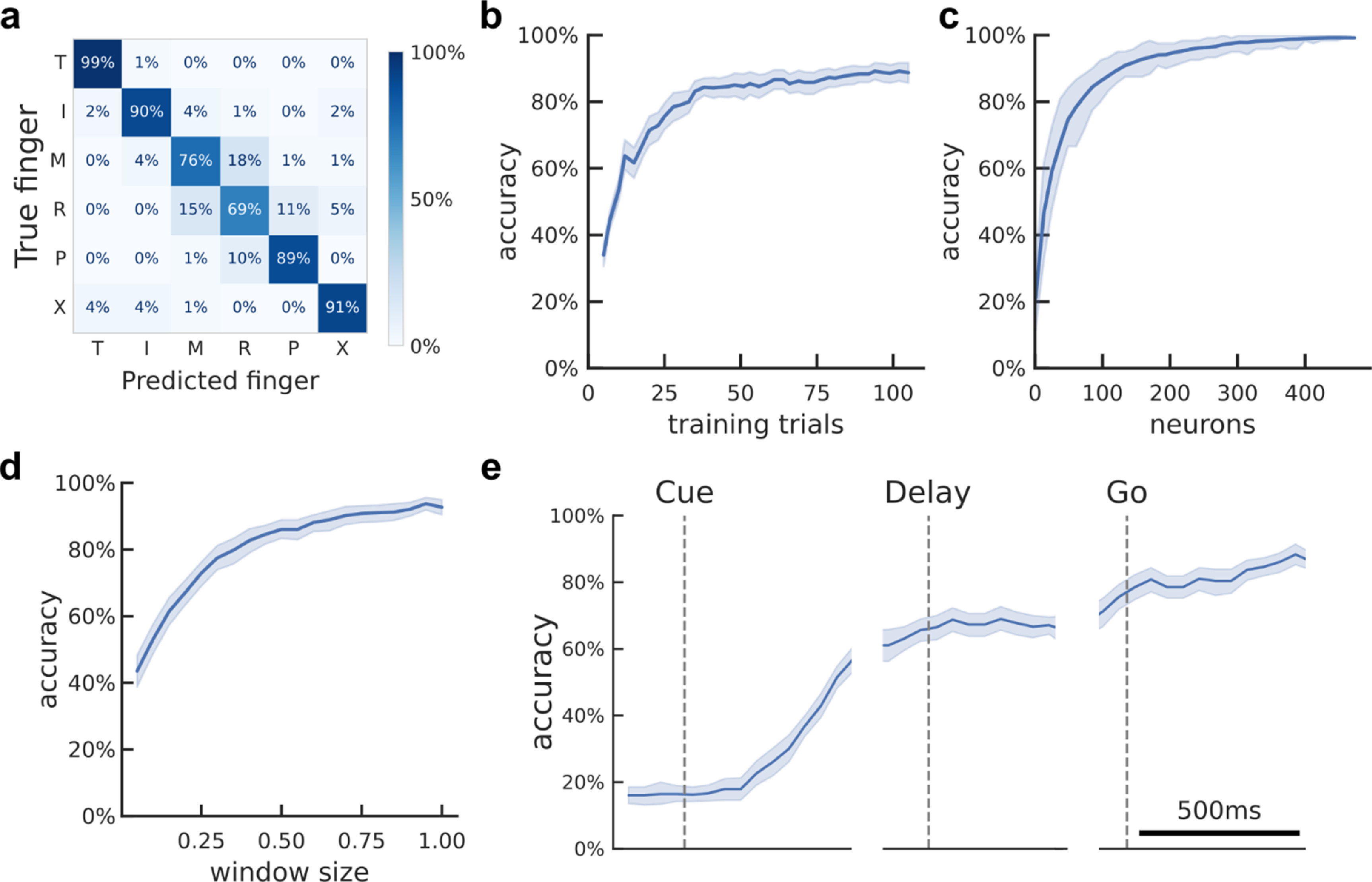
Offline classification of finger movement from population activity. (a) Cross-validated confusion matrix for classifying attempted finger movement from NS-PPC neural activity during the movement execution epoch. 86% accuracy, 480 trials over four sessions. (b) Learning curve showing cross-validated accuracy as a function of the training dataset size. About 40 trials (less than seven trials per finger) are needed to achieve 80% accuracy. Shaded area indicates 95% CI over folds/sessions. (c) Neuron-dropping curve showing cross-validated accuracy as a function of recorded neurons. Neurons were aggregated across sessions. About 70 neurons are needed to achieve 80% accuracy. Shaded area indicates 95% interval over subpopulation resamples. (d) Hyperparameter sweep showing cross-validated classification accuracy as a function of decode window size. Input features were the average firing rates in the window [200 ms, 200 ms + *window size*] after Go-cue. Window durations of about 350 ms are necessary to achieve 80% accuracy. Shaded area indicates 95% CI over folds/sessions. (e) Cross-validated classification accuracy across the trial duration (500 ms sliding window). Shaded area indicates 95% CI over folds/sessions.

Learning curves (figure 5(b)) were generated by using subsets of the training set during each stratified K-Fold split. Window duration sweeps (figure 5(d)) varied the size of the firing-rate estimation window while fixing the start time at 200 ms after the Go cue. Neural decode time-courses (figure 5(e)) used 500 ms bins centered at different times of the trial.

To visualize neuron-dropping curves (figure 5(c), supplementary figure 11), we first aggregated neurons across sessions into a pseudo-population. Specifically, we combined trials from different sessions based on their within-finger order. For example, each session’s first right-thumb trial was combined into a single trial for the pseudo-population. For the Alternating-cues finger press task with delay, participant JJ performed 96 trials in one session and 120 trials in two sessions, so we used only the first 96 trials from each session. Finally, we randomly sampled (without replacement) an *M*-neuron subpopulation from the pseudo-population. We calculated the cross-validated accuracy when decoding from this subpopulation. We varied *M* to create a neuron-dropping curve, and we repeated the subpopulation sampling 40 times for each *M* to generate 95% intervals.

### 2.8. Online BMI discrete control

Each BMI control session started with a run of the open-loop calibration task. For participant NS, this was the Alternating-cues finger press task, modified to not have a delay. For participant JJ, this was the finger press task with randomized cue location, modified to not provide classifier output.

The neural activity and finger movement cues from the calibration task served as training data for the online BMI classification model. Neural features were composed of the threshold crossing rates of each electrode during a one second window for each trial. The window start-time, ${t_{\text{s}}}$, was a hyperparameter chosen to maximize the cross-validated classification accuracy on the calibration task. The online BMI classifier was then fit to the calibration task without cross-validation. Labels consisted of the finger movement cues, and features consisted of the firing rates during each trial’s window $[{t_s},\,1 + \,{t_s}]$. Electrodes with mean firing rates <1 Hz were excluded to minimize sensitivity to discretization.

During online control of the finger grid task, the classifier predicted a single finger movement for each trial. Input neural features consisted of the threshold crossing rates from each electrode in the time window [0.5, 1.5] s after cue presentation. The BMI classifier was occasionally recalibrated between run blocks using data from this task.

As a proof-of-concept, we also connected the classifier output to the fingers of a robot hand (Shadow Dexterous Hand; supplementary video 1). On each trial, a screen cue instructed the participant which finger to press. The BMI classifier predicted each finger movement from the neural features and then moved the corresponding finger on the robotic hand.

### 2.9. Neural distance between fingers

We quantified the neural activity differences between finger movements using the cross-validated (squared) Mahalanobis distance [63]. The Mahalanobis distance is a continuous, non-saturating analogue of LDA classification accuracy [64]. Cross-validation removes the positive bias of standard distance metrics, such that $E [ {d_{jk}^2}] = 0$ when two activity patterns are statistically identical.

To calculate population distances, we used the representational similarity analysis Python toolbox [65]. The toolbox slightly modifies the cross-validated Mahalanobis equation, incorporating the noise covariances of both folds to improve robustness:

\begin{equation*}d_{jk}^2 = {\left( {{b_j} - {b_k}} \right)_{\text{A}}}{\left( {\frac{{{\Sigma _{\text{A}}} + {} {\Sigma _{\text{B}}}}}{2}{} } \right)^{ - 1}}\left( {{b_j} - {b_k}} \right)_{\text{B}}^{\text{T}}{} /{} N\end{equation*}

where ${\text{A}}$ and ${\text{B}}$ indicate independent partitions of the trials, ${{\Sigma }}$ is the noise covariance matrix, $({b_j},\,{b_k})$ are the firing rate vectors for finger movements $\left( {j,\,k} \right)$ stacked across trials, and $N$ normalizes for the number of neurons. The units of $d_{jk}^2$ are ${\text{unitles}}{{\text{s}}^2}/{\text{neuron}}$.

### 2.10. Shared representations across hands

To quantify whether finger representations were similar across hands, we compared the pairwise distances between matching finger pairs and the pairwise distances between non-matching finger pairs (figure 8(b)). We denoted a finger pair as matching if the hands differed and the finger-types were the same ([Lt, Rt], [Li, Ri], [Lm, Rm], [Lr, Rr], [Lp, Rp]). We denoted a finger pair as non-matching if the hands differed and the finger-types also differed ([Lt, Ri], [Lt, Rm], [Lt, Rr], [Lt, Rp], [Li, Rt], [Li, Rm], etc.). We described a neural population as sharing representations across hands if the average distance between matching finger pairs was smaller than the average distance between non-matching finger pairs.

### 2.11. Factorized finger representations

Factorized coding refers to representations that can be decomposed into simpler explanatory factors [66–70]. We assessed whether finger representations could be linearly decomposed into the sum of finger-type and laterality components.

We first visualized the representational geometry in figure 8(d) using 2D multidimensional scaling (MDS). MDS projects the finger movements into a low-dimensional space while attempting to preserve pairwise neural distances (figure 8(a)). We performed MDS on data from individual sessions and then used Generalized Procrustes analysis with scaling to normalize and align MDS projections across sessions. In the NS-PPC MDS plot, ellipses show standard error (S.E.) across sessions. The JJ-PPC and JJ-MC MDS plots show the mean values without any S.E. ellipses, because the two sessions with participant JJ are not sufficient to estimate the S.E.

We used leave-one-group-out cross-validation to determine whether hand- and finger-dimensions generalize to left-out movements (supplementary figure 8). If finger representations are factorized, then hand classifiers (left vs. right) should generalize when trained on a subset of finger types and evaluated on left-out finger types. Additionally, finger-type classifiers should generalize when trained on one hand and tested on the other hand (figure 8(e)). This metric is often called cross-condition generalization performance (CCGP) [70]. We pooled neurons across sessions (NS: 10 sessions; JJ: 2) into a pseudo-population. We used a permutation test to assess whether CCGP was significantly above chance, shuffling the labels repeatedly (*N* = 1001) to generate a null distribution. Standard cross-validation accuracy provides a best-case upper bound on CCGP. Reaching this upper bound implies perfect factorization. We matched training dataset sizes when comparing CCGP and within-condition cross-validation accuracy.

## 3. Results

### 3.1. Single-neuron modulation to individual finger presses

We first sought to determine whether PPC single neurons discriminate between individual finger movements. We quantified single-neuron modulation to attempted finger presses of the right (contralateral to the implant) hand while the participant performed the Alternating-cues finger press task with delay (participant NS: 120 trials per session for four sessions; participant JJ: 112 trials per session [min: 96; max: 120] for three sessions). We recorded 118 neurons per session (min: 111; max: 128) over four sessions from NS-PPC, 103 neurons per session (min: 92; max: 116) over three sessions from JJ-PPC, and 93 neurons per session (min: 90; max: 95) from JJ-MC. For each neuron, we calculated firing rates during the attempted movement period and compared firing rates across finger movements (figure 4(a), supplementary figures 2 and 3).

Similar to results from finger studies of the MC hand area [46, 50], PPC neurons were not anatomically segregated by finger selectivity. A large portion of neurons (NS-PPC: 54%; JJ-PPC: 30%; JJ-MC: 78%; figure 4(c)) varied their firing rates between attempted finger movements (*q* < 0.05), and selective neurons were often selective for multiple finger movements (mean number of significant fingers, NS-PPC: 2.1; JJ-PPC: 1.9; JJ-MC: 2.7). Moreover, many neurons discriminated between movements with large effect sizes (percentage of neurons with *η* ^2^ > 0.14, NS-PPC: 40%; JJ-PPC: 25%; JJ-MC: 64%; figure 4(d), supplementary figures 2(d) and 3(d)).

We also quantified single-neuron modulation during movement preparation. Preparatory activity discriminated between finger movements with reasonable effect sizes (figure 4(d)). Consistent with reaching studies of PPC [22], slightly fewer NS-PPC neurons had strong tuning (*q* < 0.05 and *η* ^2^ > 0.14) during movement preparation (percentage of neurons: 24%) than during movement execution (percentage of neurons: 43%) (figure 4(e)). JJ-PPC neurons modulated at similar rates during preparation (percentage of neurons with *q* < 0.05 and *η* ^2^ > 0.14: 23%) versus during execution (24%) (supplementary figure 2(e)).

### 3.2. Classifying finger presses from neural activity

Since single neurons were tuned to finger movements, we evaluated whether attempted finger movements could be classified (offline) from the population neural activity. Using data from the same task, we trained linear classifiers and assessed finger classification accuracy on held-out trials using cross-validation (methods). Classification accuracies substantially exceeded chance (accuracy, NS-PPC: 86%; JJ-PPC: 64%; JJ-MC: 84%; chance: 17%). The majority (NS-PPC: 75%; JJ-PPC: 42%; JJ-MC: 67%) of errors misclassified an adjacent finger (figure 5(a), supplementary figures 4 and 5).

Classification accuracy can depend on the neural signal quality and prediction window. To better understand how finger classification varies over dataset and classifier parameters, we quantified cross-validated accuracy across different training dataset sizes, neuron counts, and window durations (figures 5(b)–(d), supplementary figures 4 and 5).

Cross-validated accuracy increased with more training data, reaching 80% accuracy when training on about 40 trials (2.7 min) for NS-PPC. Higher neuron counts provide more finger information and thus improved classification accuracy, reaching 80% accuracy at about 70 neurons for NS-PPC. These results indicate that a single electrode array in PPC provides sufficient information to control a discrete finger-press prosthetic.

Accuracy also increased when using longer window durations, reaching 80% at durations above 350 ms. Longer window durations average out firing rates and thereby reduce the impact of measurement noise and behavioral variability on classification, but they directly mandate longer control delays. In some cases, it may be useful to minimize BMI control latency even at the expense of accuracy [71].

Finger movements could also be decoded from PPC during the planning period (figure 5(e)), although classification accuracy was lower (NS-PPC: 66%; JJ-PPC: 61%; chance: 17%) than during movement execution.

### 3.3. BMI control of finger movements

We next mapped neural activity to finger movements to control an online finger BMI, where our participants would tap each finger and their attempted movement would be decoded. For this section, we replicated a usage scenario where a prosthetic user could decide to move a finger and immediately execute the movement, without needing a delay period.

We started each session with an open-loop calibration task where the participant attempted to press fingers according to visual cues (methods). Using only a short calibration period (eight repetitions per finger, totaling about 2.5 min), each participant was able to use a classifier to accurately control individual fingers of the BMI.

**Figure 6.**
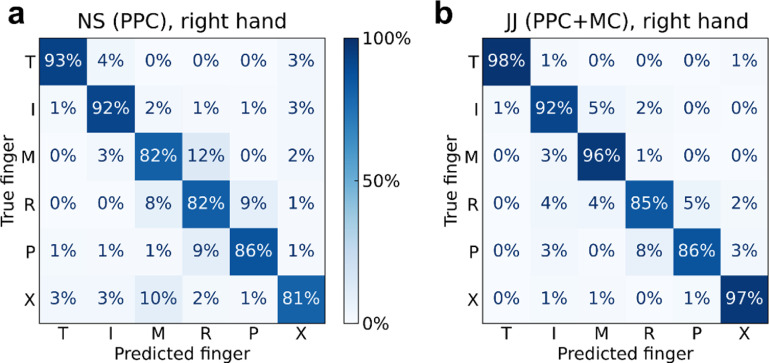
Online BMI classification of individual finger movements. (a) Confusion matrix for participant NS (PPC), right-hand finger presses. 86% accuracy ± S.D. 4% over ten sessions, 4016 total trials. Reproduced from [54]. CC BY 4.0. (b) Confusion matrix for participant JJ (PPC + MC), right-hand finger presses. 92% accuracy ± S.D. 3% over eight sessions, 1440 total trials.

The confusion matrix for participant NS (figure 6(a)) shows that she achieved high online control accuracies (86%; chance: 17%). These finger representations were robust across contexts and could be used in a range of environments. In one session, participant NS used the BMI to control the fingers of a robotic hand (supplementary video 1).

Participant JJ achieved even higher accuracies during BMI control (92% ± S.D. 3% over eight sessions; chance: 17%) (figure 6(b)). However, we note that participant JJ’s BMI decoder used threshold crossings from both MC and PPC electrode arrays, thus doubling the number of electrodes compared to participant NS. While we cannot retrospectively replicate the BMI experiment with an isolated array, we can approximate the results by training the same classification algorithm on early runs, using recordings only from a single array; we can then apply this classifier to the subsequent test trials (accuracy, JJ-PPC: 83%; JJ-MC: 87%; chance: 17%; supplementary figure 6).

On a few separate runs, participant JJ also performed the calibration and BMI control tasks with his left hand (ipsilateral to the implant). He achieved high accuracies (94% ± S.D. 4% over three sessions; chance: 17%) at a similar level to right-hand finger decoding (supplementary figure 7).

### 3.4. Classifying individual finger presses from both hands

We next investigated whether all ten finger movements could be classified from a single array. Cerebral hemispheres primarily control movement on the opposite side of the body, and we have only implanted electrode arrays in each participant’s left hemisphere. However, the ability to classify movements of both sides would reduce the number of implants necessary for bilateral BMI applications.

We examined single-neuron activity during interleaved, attempted finger presses of the contralateral (right) and ipsilateral (left) hands (methods; participant NS: 100 trials/session for ten sessions; participant JJ: 100 trials/session for two sessions). We recorded 111 neurons per session (min: 102; max: 119) from NS-PPC, 160 neurons per session (min: 159; max: 160) from JJ-PPC, and 130 neurons per session (min: 120; max: 130) from JJ-MC. Similar to the contralateral-only results, most neurons (NS-PPC: 66%; JJ-PPC: 57%; JJ-MC: 78%) discriminated firing rates across fingers (*q* < 0.05).

**Figure 7.**
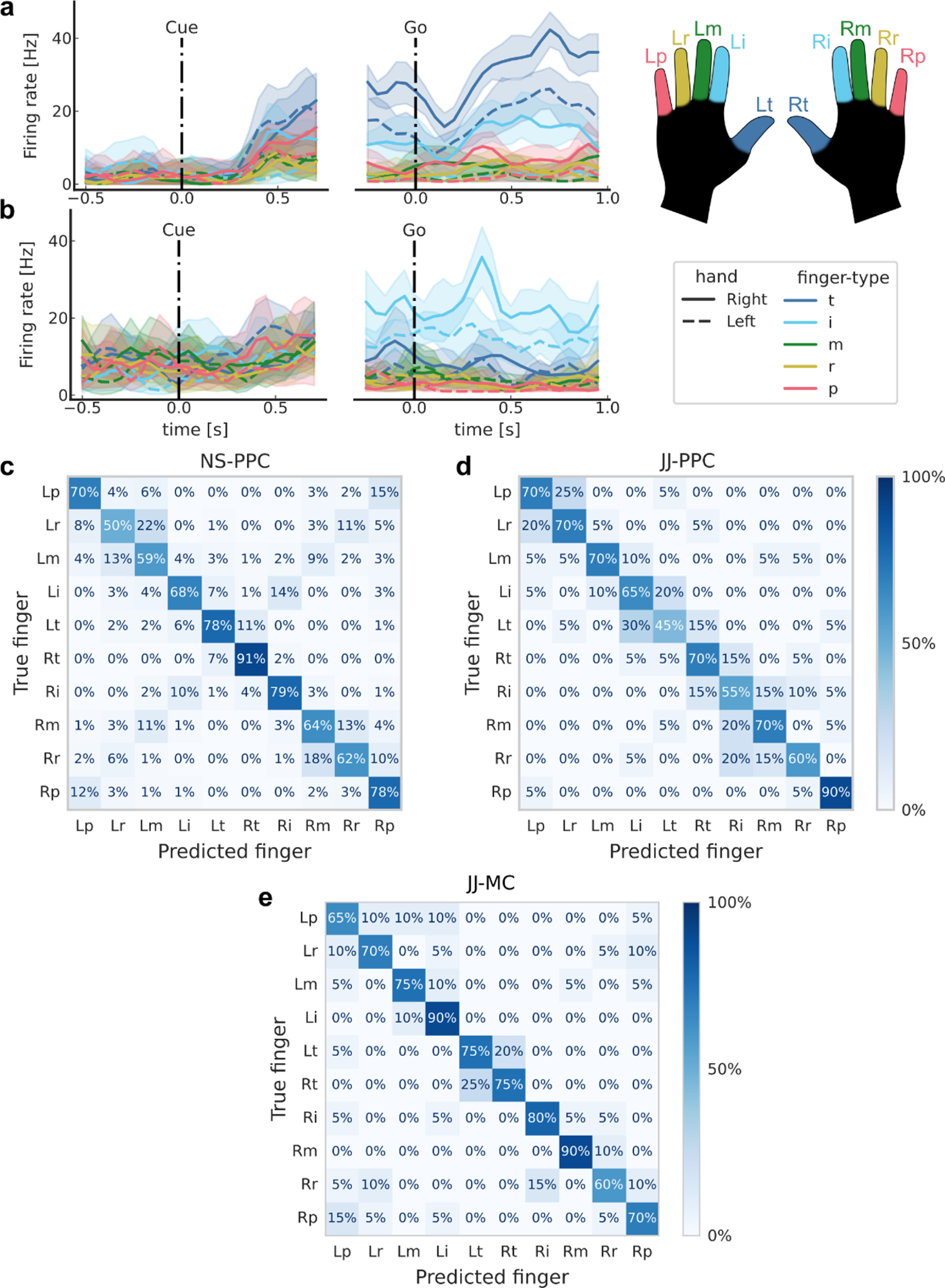
Offline classification of finger presses from both hands. (a) Mean firing rates for each finger movement for an example NS-PPC neuron, which increases its firing rate for thumb movements. Shaded areas indicate 95% confidence intervals (CI). (b) Same as (a) for a second example NS-PPC neuron, which increases it firing rate for index movements. (c) Cross-validated confusion matrix for classifying right- and left-hand finger movements from NS-PPC neural activity. 70% accuracy, 1000 trials over ten sessions. (d) Same as (c) using recordings from JJ-PPC. 66% accuracy, 200 trials over two sessions. (e) Same as (c) using recordings from JJ-MC. 75% accuracy, 200 trials over two sessions.

We then evaluated whether these signals could be used for a neural prosthetic by classifying (offline) the attempted finger movement from the population neural activity. A linear classifier (methods) was able to discriminate between all ten fingers (cross-validated classification accuracy, NS-PPC: 70%; JJ-PPC: 66%; JJ-MC: 75%; chance: 10%). The majority (NS-PPC: 76%; JJ-PPC: 66%; JJ-MC: 68%) of classification errors were adjacent-finger-confusion or matching-across-hand-confusion (figures 7(c)–(e)).

### 3.5. Factorized representation of finger type and laterality

**Figure 8.**
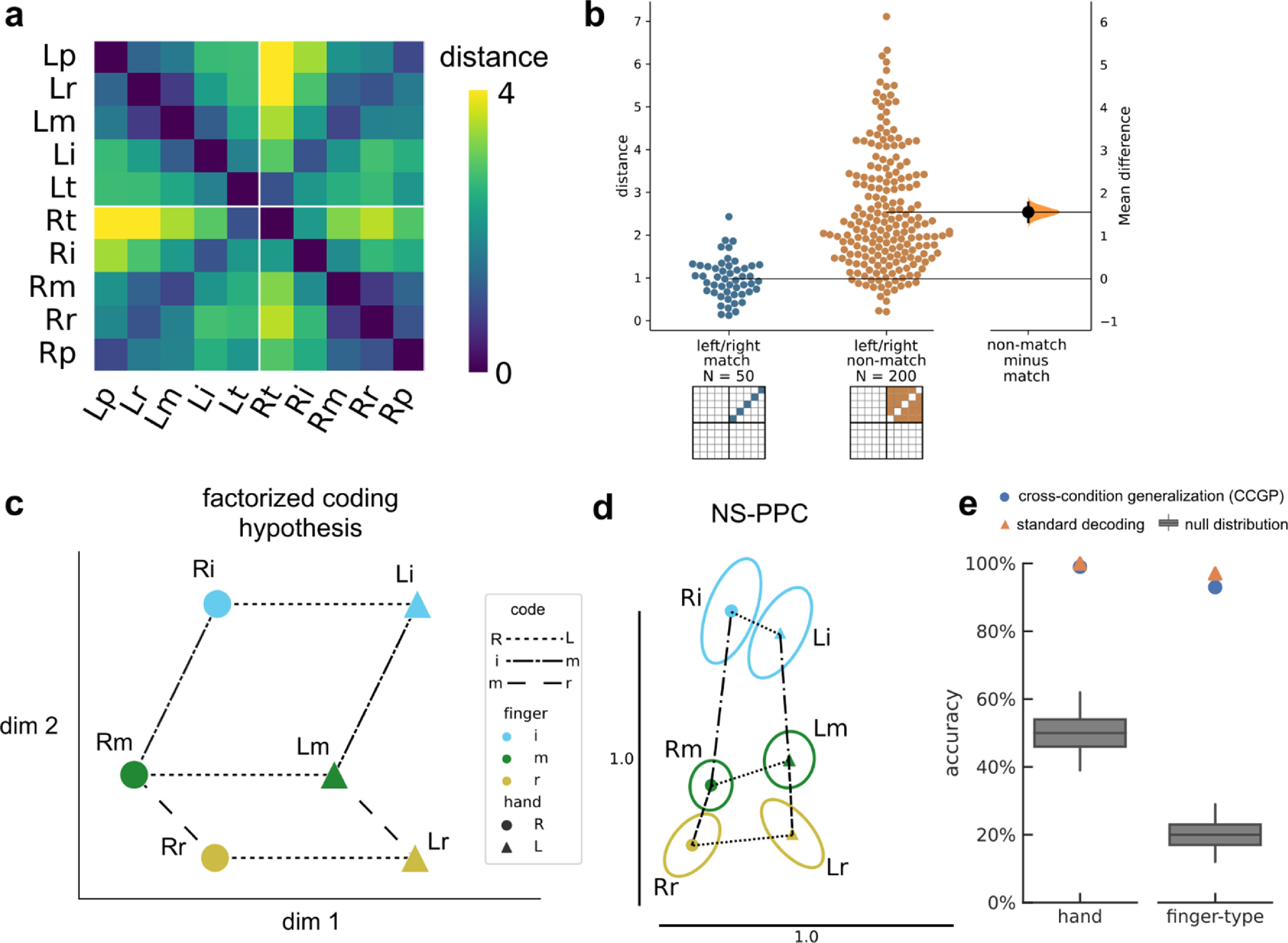
Representational geometry of contralateral and ipsilateral finger movements. (a) Cross-validated squared Mahalanobis distances between NS-PPC activity patterns during the contralateral/ipsilateral finger press task. Distances were averaged over the ten sessions. (b) Non-matching (different finger-type, different hand) finger pairs have larger distances than matching (same finger-type, different hand) finger pairs. Each circle is one element of the dissimilarity matrix of an individual session, aggregated across ten sessions. (c) Example schematic of perfect factorization along hand and finger-type components. Line styles indicate groups of parallel, identical vectors. A factorized code generalizes linearly across each component axis. For example, the Rm population activity can be constructed from the summation: Li + left→right + index→middle. For visual clarity, figure only shows three finger-types (index, middle, ring). (d) Representational geometry of finger movements corresponding to NS-PPC distances (a), visualized in 2-D using MDS. We used Generalized Procrustes analysis (with scaling) to align across ten sessions. Ellipses show S.E. across sessions. Scale bars shown. Vectors with matching line-styles match each other, suggesting that the neural code is factorized. (e) Linear decoders generalized (supplementary figure 8) across finger-type to classify hand (left) and across hand to classify finger-type (right) (*p* < 0.001, permutation test), indicating that movement representations were factorized across finger-type and hand dimensions.

To characterize how NS-PPC simultaneously represents contralateral and ipsilateral finger movements, we calculated the cross-validated neural distances between pairs of attempted finger movements. Figure 8(a) visualizes these distances in a representational dissimilarity matrix [55] that is row- and column-indexed by finger. Visual inspection shows that neural distances are small between right/left pairs of fingers (anti-diagonal of figure 8(a)), suggesting that movement representations are partially shared across hands. On average, matching right/left finger pairs were 1.56 distance-units (95% CI: [1.33, 1.78], figure 8(b)) closer to each other than non-matching fingers were. Matching fingers were also represented more similarly than non-matching fingers in JJ-MC (mean difference: 4.30, 95% CI: [2.74, 5.46], supplementary figure 9(b)), but this result was not conclusive in JJ-PPC (mean difference: 0.27, 95% CI: [–0.17, 0.64], supplementary figure 10(b)).

What representational geometry allows downstream readout of all ten fingers (figure 7) while sharing information across hands (figure 8(b))? Studies of human MC [72–75] have also found correlated representations across sides, with [73] linearly decomposing population activity into simpler factors: laterality, arm-versus-leg, and motion pattern.

Do laterality and finger-type also form a factorized code in PPC and MC? In a perfectly factorized representation (figure 8(c)), vectors between neural representations are simply the summation of the vectors between their respective components. For example, the vector Lm→Ri can be decomposed into generic left→right and middle→index vectors. Geometrically, these generic vectors would form parallelograms between relevant groups of conditions (figure 8(c)) [76]. In other words, a factorized code would have a consistent hand subspace and a consistent finger-type subspace, although these subspaces need not be orthogonal.

We used 2D MDS to visualize the geometric relationship between NS-PPC finger representations (figure 8(d)), limiting to the index, middle, and ring fingers for visual clarity. We found that inter-finger vectors were similar across hands, with the index finger relatively distinct from the middle and ring fingers, consistent with previous studies of contralateral finger movements [54, 77]. Additionally, the left→right vector appeared identical across all matching left/right finger pairs.

Factorized coding generalizes across the axes of the simpler building blocks. Since individual left→right vectors are nearly identical to each other, linear decoders trained to differentiate left-vs-right on a subset of finger types (Lt-vs-Rt; Li-vs-Ri, Lm-vs-Rm, Lr-vs-Rr) should generalize to held-out, hypothetically equivalent vectors (Lp-vs-Rp) (supplementary figure 8). We aggregated neurons across different sessions into a pseudo-population (methods). Consistent with the factorized coding hypothesis, cross-condition hand-decoding generalization performance (hand CCGP) was nearly perfect (accuracy using 1111 neurons: 99%, chance = 50%, *p* < 0.001, permutation test). Next, we applied cross-decoding to the finger dimension, training a classifier to discriminate between fingers of the right hand and then testing on the left hand (and vice-versa). The finger-type dimension also generalized well across hands (accuracy: 93%, chance = 20%, *p* < 0.001), and finger-type CCGP was close to the standard cross-validation accuracy (98%) evaluated using within-condition cross-validation (figure 8(e)); this within-condition cross-validation accuracy is a best-case upper bound on CCGP. The close match between finger-type CCGP and cross-validation accuracy indicated that the finger-type dimension robustly generalized across hands. This result demonstrates that NS-PPC finger representations can be decomposed linearly into hand and finger-type building blocks.

Comparable results held for JJ-MC recordings, with robust factorization of the neural code into hand and finger-type components (hand CCGP using 259 neurons: 86%, chance = 50%, *p* < 0.001; standard hand cross-validation accuracy: 87%) (finger-type CCGP: 75%, chance = 20%, *p* < 0.001; standard finger-type cross-validation accuracy: 89%) (supplementary figure 9). Interestingly, JJ-PPC finger representations were less factorized. While above chance (*p* < 0.001), the finger-type CCGP (36%, using 319 neurons) was much lower than the within-condition cross-validation accuracy (65%) (supplementary figure 10). Even when accounting for differences in neural population size, finger-type CCGP for JJ-PPC was lower than finger-type CCGP for NS-PPC and JJ-MC (supplementary figure 11).

## 4. Discussion

Human dexterity is characterized by our ability to quickly reach-and-grasp, as well as our ability to move individual fingers volitionally beyond basic grasp templates [9]. Individual finger movements are generally considered to be the domain of the MC hand knob, while the PPC complements via higher-level computations, such as transforming object shape to grip type [42]. This perception is supported by a wide range of evidence [8]; for example, fMRI studies find topographic finger activation maps in MC [77, 78] but not in PPC [79]. Despite the lack of coarse finger topography in PPC, here we found that neurons in two grasp-related regions of PPC were discriminative for attempted finger movements. Population tuning was robust enough for human participants to control finger BMIs in a variety of applications. These results demonstrate that detailed information about finger movements is more distributed than is commonly thought.

Our study adds to a growing number of finger BMI demonstrations. Previously, [34] demonstrated the first online neural decoding of all-five individual finger movements in human participants, using a high-density ECoG grid over the sensorimotor cortex. Similar to our study [36], implanted intracortical arrays in the MC of a tetraplegic participant and decoded attempted finger movements, achieving an offline accuracy of 67%. Recently [33, 49], achieved high-performance continuous control of flexion and extension of two finger groups. Our results contribute to prior studies by showing that simultaneous PPC + MC recordings can improve online finger decoding accuracies (figure 6). Considering that PPC and MC usually fulfill different functions for able-bodied sensorimotor control [8], an interesting future direction will be to understand to what degree PPC and MC complement each other across more diverse BMI control paradigms.

Algorithmic advances may further improve finger decoding performance. For example, hierarchical classifiers might be useful for classifying finger direction and finger movement [34]. Additionally, with larger data quantities or with data augmentation strategies, time-varying and nonlinear classifiers like recurrent neural networks can improve neural decoding [32, 49, 80, 81]. Performance improvements may also come from decoding non-traditional variables, such as handwriting [32] or goals [22]. State-machine control (common in other assistive technologies like myoelectric prostheses [82] or Dwell) and AI-assisted hybrid control [83, 84] may further improve BMI usability. In combination with somatosensory intracortical microstimulation to generate fingertip sensations [47, 48], such methods could enable a functional hand prosthetic.

After demonstrating BMI control of the contralateral fingers, we studied representations of ipsilateral finger movements. We found that a linear classifier could discriminate between movements of all ten fingers (figure 7). Given that descending corticospinal tracts primarily cross to control the contralateral side, it was interesting to find that ipsilateral finger decoding was relatively robust. On some sessions, ipsilateral decoding accuracies were even comparable to contralateral decoding (supplementary figure 7). The strong ipsilateral coding found here differs slightly from fMRI studies, which find that ipsilateral finger coding is about a quarter of the strength of contralateral finger coding [74, 85]. Intracortical electrophysiology studies of ipsilateral grasping and arm movements find a stronger range of ipsilateral coding; ipsilateral coding strength varies from ∼40% [72, 86] to >80% [72, 73, 86, 87] of the contralateral coding strength in MC, depending on the subject and the specific metric compared. To better understand the role of ipsilateral finger activity, future single-neuron studies could investigate how individual finger representations mix to construct multi-finger movements, both within and across hands. fMRI studies of sensorimotor cortex suggest that same-hand movements would be organized by their natural usage patterns [77], while both-hand movements would exclusively represent the contralateral fingers [74]. An open question is whether these patterns also extend to single-neuron populations and to PPC.

Even as the ten finger movements were discriminable, activity patterns for NS-PPC and JJ-MC were similar across corresponding finger pairs on opposite hands (figures 8(a) and (b)). Our results match other studies that have also found shared-yet-separable hand representations in macaque anterior intraparietal area [88] and human MC [73, 74]. This pattern of cross-condition generalization has previously been described as partially mixed selectivity [43], abstract or factorized representations [70], or compositional coding [66, 73]. Here, the NS-PPC and JJ-MC finger codes could be factorized into finger-type and laterality subspaces (figures 8(d) and (e)), resembling the partial compositionality described by [73] for arm and leg movements. Compositional and factorized coding have been speculated to play a number of different computational functions, from skill transfer to general cognition [43, 66, 67, 72, 73]. For neuroprosthetic applications, factorized coding simplifies decoder calibration. Because neural coding generalizes across conditions, decoders can train on only the underlying factors, rather than every combination.

Surprisingly, JJ-PPC population activity was not factorized to the same extent as NS-PPC and JJ-MC. The difference between JJ-PPC and NS-PPC results might stem from neuroanatomical variability [89, 90] or differences in implant location. The NS-PPC implant was located at the junction of the postcentral and intraparietal sulci (PC-IP), an area involved in grasping and fine finger movements [4, 89, 91]. PC-IP receives inputs from neighboring somatosensory cortex [90, 92], suggesting that it may facilitate state estimation of the hand [54, 93, 94]. We could not implant the JJ-PPC recording array in the center of the PPC grasping area, functionally localized near PC-IP (supplementary figure 1), because blood vessels obstructed the cortical surface. Thus, we implanted the JJ-PPC array in the SPL, medial and posterior compared to the NS-PPC implant. Medial and posterior areas of PPC tend to receive stronger visual inputs [90, 92, 95] and are more involved in reaching than grasping [96], so the recorded JJ-PPC population could be more involved in calculating visuomotor transforms [92, 97] for visually guided reaching [90, 96]. It is possible that the difference in implant location also contributed to differences in contralateral finger tuning between NS-PPC (figure 4) and JJ-PPC (supplementary figure 2). However, it is difficult to precisely compare implant locations, because the anatomical location of individual functional areas can vary widely between participants [89, 90]. Future comparisons may benefit from multi-modal preoperative neuroimaging to map implant locations onto standard parcellations [98].

## 5. Conclusions

The PPC has long been known to be involved in the reaching and grasping of objects, but less is known about its contribution to individual finger movements. Here, two tetraplegic participants controlled individual fingers through BMIs recording from the PPC and MC. Ipsilateral finger coding was strong in all three recorded neural populations, and two of the populations exhibited factorized coding that enabled decoders to simultaneously generalize across and discriminate between hands. Our results demonstrate that PPC and MC can provide complementary control signals for assistive neuroprosthetics.

## Data Availability

The data that support the findings of this study are openly available at the following URL: https://dandiarchive.org/dandiset/000252.
